# The impact of the genotype on the prevalence of classical scrapie at population level

**DOI:** 10.1186/1297-9716-42-31

**Published:** 2011-02-15

**Authors:** Angel Ortiz-Pelaez, Juana Bianchini

**Affiliations:** 1 Centre for Epidemiology and Risk Analysis, Veterinary Laboratories Agency, Woodham Lane, Weybridge, UK

## Abstract

Total number and genotypes of animals in holdings selected for the genotype & cull option in the Compulsory Scrapie Flock Scheme (CSFS) in Great Britain were extracted from the National Scrapie Plan data warehouse. The association between various genotype-related measures and scrapie prevalence infection was tested using zero-inflated negative binomial models with the counts of positive cases as dependent variable, and country, number of flocks in the scheme, flock size, surveillance source and the following genotype-related measurements: the centered-log ratios (*clr*) oof the 15 genotypes, of the proportions of the 5 alleles at codons 136, 154 and 171, of the proportions of the 5 NSP types, and two flock-susceptibility risk indicators, as explanatory variables. A total of 319341 genotyped animals from 168 holdings were included in the analysis. An increased proportion of the ARR/ARR genotype corresponded to a decrease in the number of scrapie cases. ARR/AHQ, AHQ/VRQ, ARH/VRQ and ARQ/VRQ genotypes, NSP type V, ARH, ARQ, AHQ and VRQ alleles and the low and high-susceptibility risk indicators are all associated with an increase risk in the number of scrapie cases.

Regardless the management practices; the increased susceptibility that the non-ARR alleles confer on an individual could be extrapolated at the population level. Increasing prevalence of ARR allele reduces the overall risk of scrapie at population level. At genotype level, the VRQ/VRQ genotype, present a very low frequency in the study population, seems to play a residual effect in the overall risk of scrapie in a flock.

## Introduction

Scrapie is a transmissible spongiform encephalopathy (TSE) affecting small ruminants. It is an endemic disease in Great Britain, which only became notifiable in 1993, in accordance with EU Council Directive 91/68/EC. The genetic susceptibility of sheep to classical scrapie has been the backbone of the breeding for resistance programmes successfully implemented in the EU as per EC Regulation 999/2001. In the British sheep population, the polymorphisms of the ovine *PRNP *gene at codons 136, 154 and 171 determine the presence of five possible alleles: ARR, AHQ, ARH, ARQ and VRQ. Numerous studies have addressed the different levels of susceptibility/resistance to infection and clinical disease [1-3] and the length of the incubation period that the 15 naturally occurring different combinations of alleles confer to the individual animal [4]. Results of the attack rates and number of reported and tested cases in the population of each genotype have been reported elsewhere [5,6].

The risk of infection or clinical classical scrapie at population level has been studied much less frequently given the difficulties of selecting and following up scrapie-affected flocks over time. Some attempts have been made with differing levels of success, but most of them were subject to bias in the genotype data sources or in the selection of flocks for study, mainly managed by research institutes [7]. In some cases, it was unavoidable to prevent farmers from altering their breeding policy once the genotype of the animals had been revealed [8]. The relationship between genetic, phenotypic factors and scrapie has been studied in some individual flocks in Great Britain during the last ten years. Descriptions of scrapie epidemics in flock with known genotypes are available in the literature like the scrapie epidemic in a Texel sheep flock [9] and in a closed flock of Romanov sheep [10]. Tongue et al. [11] reported genotype distributions of 15 scrapie-affected flocks in Great Britain. Baylis et al. [5] described the greater scrapie risk for the ARQ/VRQ, ARH/VRQ and VRQ/VRQ genotypes and the resistance conferred by the ARR allele when comparing surveillance scrapie data and the genotypes from 44 flocks used in research studies. McIntyre et al. [8] described the genotype profile of 30 naturally-infected flocks with classical scrapie with cases only present in animals possessing at least the ARQ or VRQ alleles. Increased risk of scrapie has been associated with high frequencies of the ARR/VRQ genotype, with a VRQ frequency of more than 5.2% or with VRQ/VRQ and AHQ/VRQ at any frequency [12]. More recently the association between genotype and breed with the risk of classical scrapie was studied crossing data from available databases on clinical cases, genotypes and breeds in the UK [13].

The individual association between genotype and risk of scrapie may not be mirrored at the population level given the interaction between the genetic susceptibility of a number of individuals, the strain of the scrapie [4] and environmental factors [14], so that the additive effect of the individual risk for all animals in a flock does not equal the overall risk of the flock to scrapie. The availability of genotype data from scrapie-affected flocks allows the determination of the overall risk of scrapie using the complete genotype profile of a flock and the frequency of scrapie, measured by the prevalence of the disease during the implementation of statutory eradication measures under the Compulsory Scrapie Flocks Scheme (CSFS). Details of this scheme have been described elsewhere [15]. The objective of this study was to determine whether individual risk correlates with risk at the holding level by testing the association between the frequency of the different genotypes expressed by several genotype-related measures and the prevalence of scrapie infection defined as the presence of PrP^Sc ^in the obex at detectable levels by approved diagnostic tests used in surveillance with or without the presence of clinical disease (clinical status is not ascertained at the time of the cull) in holdings affected with scrapie.

## Materials and methods

Genotype data and the total number of animals in holdings that were selected for the genotype & cull option in the Compulsory Scrapie Flock Scheme (CSFS) in Great Britain between 1 April 2005 to 31 December 2007 were extracted from ARCADIA, the data warehouse of the National Scrapie Plan (NSP). This option includes the genotyping and selective cull (initial cull) of Type 3 and 5 genotypes in ewes and non-Type 1 genotypes in rams, with Type 4 genotype ewes being allowed to be slaughtered for human consumption. The 5 types defined by the NSP are: Type 1 (ARR/ARR), Type 2 (ARR/AHQ, ARR/ARH, ARR/ARQ), Type 3 (AHQ/AHQ, AHQ/ARH, AHQ/ARQ, ARH/ARH, ARH/ARQ, ARQ/ARQ), Type 4 (ARR/VRQ) and Type 5 (AHQ/VRQ, ARH/VRQ, ARQ/VRQ, VRQ/VRQ). They establish decreasing levels of resistance to classical scrapie with ARR/ARR or Type 1 being the most resistant and genotypes with alleles VRQ and non-ARR, the Type 5, the least resistant. Genotypes were obtained from blood samples taken once the holding had joined the CSFS under the genotype and cull option.

Sheep with a date of death or slaughter on or after the date of the initial cull were removed. The number of confirmed cases of classical scrapie, based on a sample of the cull population of sheep older than 12 months of age, was extracted for each study holding from the results of the testing of the initial cull stored in the CSFS database, maintained at the Veterinary Laboratories Agency (VLA). All sampled animals were tested using the Bio-Rad TeSeE ELISA as a screening test. If positive, Immunohistochemistry (IHC) and VLA Hybrid Western Blot were used to test samples of obex and/or cerebellum to confirm the case and to discriminate between classical and atypical scrapie [16]. Additional attribute data at the holding level, namely, number of flocks in the scheme, surveillance source of the index case and country were available from a previous analysis [15]. ANOVA test assuming equal variances was applied to compare mean proportions of genotypes across categories.

Numeric covariates with the proportion of animals of each genotype, NSP type and allele were extracted. They represent parts of the total distribution of the three genotype-related variables in the study holdings whereby their values add up to 1 for each, in what is called compositional data. This set of variables in a dataset is not free to vary independently. Since the genotype covariates for each observation add up to a constant 100%, traditional statistical analysis cannot be applied as they can induce spurious correlations [17]. In order to overcome this structural characteristic of the dataset, the centred-log-ratio (*clr*) transformation was applied to the compositional dataset, as described by Aitchison [18]. Each value in the set of the compositional data is replaced by an alternative value. For an *i-part *composition *x *= [*x*_1_,*x*_2_,...,*x_i_*] where *i *is the number of covariates in the set of compositional data (fifteen in the genotype covariates and five in both the NSP type and allele covariates) and *x_i _*the value of each covariate (proportion of animals), the centred-log-ratio (*clr*) transformation is calculated following equation (1)

(1) clr(x)=clr[xi]clr[xi]=[ln⁡xi(∏xi)1i]⋅

To avoid zero values in the denominator of equation (1), observations with no animals of certain genotype, NSP type or allele were replaced by 0.01 and the corresponding variable with the maximum value was reduced by the same amount [19].

The dependent variable was defined as the number of positive cases confirmed in the initial cull. Four explanatory variables were included in the final dataset for analysis: country (England/Wales/Scotland), number of flocks in the scheme (numeric), total number of animals genotyped available before the initial cull (numeric) and the surveillance source of the index case (abattoir survey/fallen stock/clinical suspect). An additional explanatory variable was included in each of the three models: the *clr *of the proportions of the 15 genotypes (model 1), the *clr *of the proportions of the five NSP types (model 2) and the *clr *of the proportions of the five alleles (model 3).

Given the large proportion of the holdings with no positive cases confirmed in the initial cull (113, 67.2%) and the over-dispersion of the distribution of counts of scrapie cases, shown by the standard deviation being twice the mean (mean = 0.8 standard deviation = 1.7), linear models with a zero-inflated negative binomial distribution with robust standard errors were fitted.

A manual backwards stepwise variable selection was applied to the full models. Linear combination of continuous variables was tested by transformation into quartiles categories or discretional groups, except for the *clr*-transformed covariates. Due to the small sample size and the large number of variables, once the main effects which significantly affected the dependent variable had been established, the significance of pairwise interaction terms were tested. Fitness of the models was assessed using the Akaike Information Criterion (AIC) and the deviance of the models. The Vuong test [20] was applied to assess whether zero-inflated models better fit the data than negative binomial regression models.

A further analysis to assess the effects of the flock-level susceptibility was conducted using two flock-susceptibility risk indicators, *s_risk _*[8]. Both indicators are based on the relative frequency of genotypes in the flock weighted according to the risk of scrapie in that genotype, so that,

$$s_{risk}=\sum_{j}r_{j}f_{j}$$

where *f_j _*is the proportion of the flock of genotype *j *and *r_j _*is the estimate for risk of scrapie in that genotype relative to VRQ/VRQ, The risk estimates, *r_j_*, were derived from two sets of genotype frequencies at national level producing two estimates: low (model 4) and high (model 5) susceptibility [21]. Both high and low-susceptibility risk indicators were calculated using the proportion of genotypes in each study holding and tested using the statistical models as above described. All the analysis was conducted using Stata© 10 (StataCorp. 2007. Stata Statistical Software: Release 10. College Station, Texas, USA).

## Results

One hundred and sixty-eight holdings were culled using the genotype & cull option: 57 (34%) in England, 86 (51%) in Wales and 25 (15%) in Scotland. Most of the holdings (73%) had one or two flocks in the scheme with only 17 (10%) having four or more. In terms of the surveillance source of the index case, 121 holdings (72%) were identified as scrapie-infected by passive surveillance, 39 (23%) by surveillance of fallen stock/dead-in-transit cases and 8 (5%) by the abattoir survey. The total number of genotyped animals included in the dataset was 319 341 (mean per holding: 1 900). The ARR/ARR was the most frequent genotype (30.8% of all animals) and had the highest mean proportion across the holdings of the study population (31.2%), followed by ARR/ARQ (27.7%) and ARR/AHQ (10.8%). At the other extreme AHQ/ARQ, ARH/ARH, ARH/VRQ and VRQ/VRQ were the least frequent genotypes (mean proportion of 0.5% or less) (see Table 1).

**Table 1:** General statistics of the proportion of genotypes, NSP types and alleles in the 168 holdings included in the study

	Mean %	Median %	Standard Deviation %	Minimum %	Maximum %
**Genotypes**					
ARR/ARR	31.2	31.5	11.8	0.0	72.0
ARR/ARQ	27.7	27.7	8.5	0.0	58.8
ARR/AHQ	10.8	10.2	6.7	0.0	39.1
ARR/ARH	3.3	1.1	4.7	0.0	21.4
AHQ/AHQ	1.2	0.7	1.7	0.0	12.3
AHQ/ARH	0.5	0.1	1.1	0.0	6.5
AHQ/ARQ	4.9	4.6	3.5	0.0	18.2
ARH/ARH	0.5	0.0	2.3	0.0	28.6
ARH/ARQ	1.5	0.4	2.4	0.0	12.2
ARQ/ARQ	9.0	6.6	11.4	0.0	94.7
ARR/VRQ	4.1	3.6	3.7	0.0	27.7
AHQ/VRQ	1.1	0.9	1.1	0.0	4.7
ARH/VRQ	0.4	0.0	1.3	0.0	14.3
ARQ/VRQ	3.2	2.4	3.4	0.0	22.1
VRQ/VRQ	0.3	0.1	0.7	0.0	5.4
					
**NSP types**					
Type I	31.2	31.5	11.8	0.0	72
Type II	41.8	42.3	9.3	0.0	68.1
Type III	17.8	15.6	12.6	0.0	94.7
Type IV	4.1	3.6	3.7	0.0	27.7
Type V	5.1	4.3	4.6	0.0	31
					
**Alleles**					
ARR	54.1	55.4	12.8	0.1	85.9
ARH	3.5	1	5.9	0.02	49.9
ARQ	27.7	25.6	12.4	6	97.1
AHQ	10	9.3	5.9	0.1	33.7
VRQ	4.7	4	3.6	0.1	29.5

**Figure 1:**
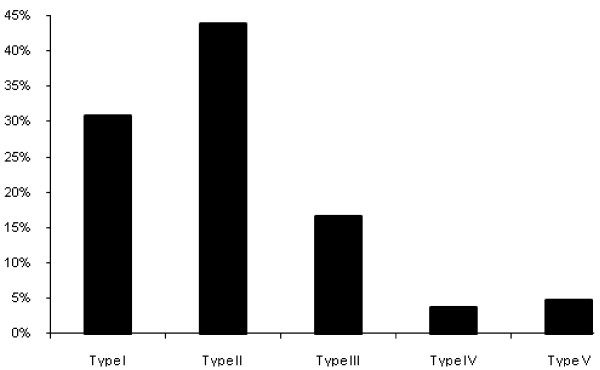
Proportion of sheep in the study holdings with genotypes according to the NSP types.

NSP type II was the most frequent (43.8% of all animals) and a mean proportion in all holdings of 41.8%, followed by type I, type III, type V and then type IV in decreasing order of frequency (Figure 1 and Table 1).

**Figure 2:**
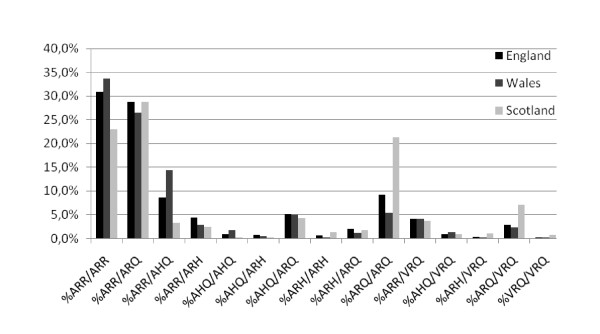
**Distribution of average proportion of genotypes in the 168 holdings included in the study by country**.

By country there were some differences in the distribution of certain genotypes. Holdings in Scotland had on average 23% of ARR/ARR animals compared to 30.8% in England and 33.7% in Wales. The largest difference between countries was that of the ARR/AHQ allele with 3.3% in Scotland, 8.6% in England and 14.4% in Wales, and that of ARQ/ARQ, much more frequent in Scotland (21.2%) than in England (9.1%) and Wales (5.4%). The proportion of the ARQ/VRQ genotype in Scottish holdings (7.1%) was higher than in England (2.9%) and Wales (2.2%) (Figure 2). Significant differences at the 5% level (ANOVA test assuming equal variances) in the proportion of animals across countries were found in these four genotypes.

During the initial cull 138 cases of classical scrapie were confirmed in the study holdings from a total of 19 666 tested animals (mean: 117. STD: 61,6. Range: 1-304) with an overall prevalence of 0.7% (95% CI: 0.58-0.82). Only 55 holdings (32.7%) had positive cases with a mean prevalence among positive holdings of 1.9% (range: 0.4-8.6%) whereas the remaining 113 holdings had no positive cases in the initial cull.

The final model 1 included *clr *of ARR/ARR, ARR/AHQ, AHQ/VRQ, ARH/VRQ and ARQ/VRQ, country and number of flocks. The predicted number of cases increased along with the increase in the *clr *of ARR/AHQ, AHQ/VRQ, ARH/VRQ, ARQ/VRQ; decreased with the *clr *of ARR/ARR and was greater in Wales (*P *< 0.01) compared to the baseline (England). The *clr *of AHQ/VRQ was marginally significant (*P *= 0.051) and was left in the model. The increase in the number of flocks in the scheme increased the number of cases of scrapie. The interaction between country and number of flocks was significant for Wales where the predicted number of cases decreased non-linearly with an increase in the number of flocks in Wales.

The final model 2 included *clr *of NSP type V, country and number of flocks. The predicted number of cases increased along with the increase in the *clr *of NSP type V. The expected number of cases was greater in Wales (*P *< 0.01) compared to the baseline (England). Having four or more flocks in the scheme increased the number of cases of scrapie (*P *= 0.02). The interaction between country and number of flocks was significant for Wales where the predicted number of cases decreased non-linearly with the increase in the number of flocks in Wales.

The final model 3 included *clr *of ARH, ARQ, AHQ and VRQ, country, holding size and number of flocks. The predicted number of cases increased along with the increase in the *clr *of ARH, ARQ, AHQ and VRQ alleles. The expected number of cases was greater in Wales (*P *< 0.01) compared to the baseline (England). Having four or more flocks in the scheme increased the number of cases of scrapie (*P *= 0.01). If the total number of animals was between 300 and 1 000 or greater than 1 000, the change in log (count of scrapie cases) was similar: 1.5. The interaction between country and number of flocks was significant for Wales where the predicted number of cases decreased non-linearly with the increase in the number of flocks in Wales.

In all three models the predictors of excess zeros, *clr *variables, were not statistically significant and the Vuong test (*P *< 0.05) suggested that the zero-inflated model was a significant improvement over a standard negative binomial model. Coefficients of the model, incidence rate ratios and 95% confidence intervals for the final three models using the centred-log-ratio (*clr*) transformations are shown in Table 2.

**Table 2:** Coefficients, incidence risk ratios (IRR) and their 95% confidence intervals for the final three models using the count of positive confirmed cases in the initial cull as dependent variable and the centred-log-ratio (clr) transformations of the proportions of genotypes, NSP types and alleles.

Variables	Model 1 (Genotypes)			Model 2 (NSP types)			Model 3 (alleles)		
	**Coef**.	**IRR**	**95%CI**	**Coef**.	**IRR**	**95%CI**	**Coef**.	**IRR**	**95%CI**
constant	0.7		-0.5-1.9	-0.7		-2.1-0.7	-0.3		-2-1.3
									
clr % ARR/ARR	**-0.5**	**0.5**	**0.4-0.8**						
clr % ARR/AHQ	**0.5**	**1.7**	**1.2-2.2**						
clr % AHQ/VRQ	0.2	1.2	0.99-1.5						
clr % ARH/VRQ	**0.3**	**1.3**	**1.08-1.7**						
clr % ARQ/VRQ	**1.7**	**1.9**	**1.5-2.6**						
									
clr % NSP V				**1.2**	**3.4**	**2.2-5.2**			
									
clr % ARH							**0.8**	**2.3**	**1.2-4.4**
clr % ARQ							**1.3**	**3.7**	**1.2-11.6**
clr % AHQ							**1**	**2.7**	**1.2-5.9**
clr % VRQ							**1.4**	**4**	**2-8**
									
Country									
Wales	**1.7**	**5.8**	**2.3-14.9**	**1.6**	**5**	**2.1-12.1**	**1.5**	**4.4**	**1.8-10.9**
Scotland	-1.1	0.3	0.08-1.4	0.8	2.3	0.3-17.8	1.2	3.4	0.6-20.8
									
# flocks (baseline: 1)									
2	0.9	2.6	0.9-7.7	0.7	2	0.8-5.4	0.7	2	0.7-5.4
3	0.7	2	0.6-6.4	0.6	1.8	0.6-5.5	0.5	1.7	0.5-5.3
4 or more	**1.7**	**5.4**	**1.9-15.6**	**1.4**	**3.9**	**1.4-10.7**	**1.3**	**3.8**	**1.3-11.3**
									
# animals (baseline: < 300)									
300-1000							**1.5**	**4.3**	**1.2-16.1**
> 1000							**1.5**	**4.7**	**1.3-16.7**
									
Wales × 2 flocks	**-1.9**	**0.1**	**0.04-0.5**	**-1.5**	**0.2**	**0.07-0.7**	**-1.3**	**0.2**	**0.08-0.8**

Parameters in bold are significant at 0.05 level

The increase in both low and high-susceptibility risk indicators increased the expected number of scrapie cases. In both models the expected number of cases was greater in Wales (albeit marginally in model 4) and in Scotland, compared to the baseline (England), and if the total number of animals was greater than 300. The interaction between country and number of animals was significant for Scotland where the predicted number of cases decreased non-linearly with the increase in the number of animals in holdings in Scotland. Coefficients of the models, incidence rate ratios and 95% confidence intervals for the final two models using the flock-level susceptibility risk indicators are shown in Table 3.

**Table 3:** Coefficients, incidence risk ratios and 95% and their confidence intervals for the final two models using the count of positive confirmed cases in the initial cull as dependent variable and the flock-susceptibility risk indicators as the main explanatory variables.

Variables	Model 4 (Low susceptibility)			Model 5 (High susceptibility)		
	**Coef**.	**IRR**	**95%CI**	**Coef**.	**IRR**	**95%CI**
constant	-4.05		-6.2- -1.91	-4.23		-6.16- -2.3
						
Flock-susceptibility risk indicator	**0.42**	**1.52**	**1.15-2**	**0.46**	**1.59**	**1.21-2**
						
Country						
Wales	1.93	6.9	0.8-60	**2.2**	**9**	**1.12-72.2**
Scotland	**2.24**	**9.4**	**1.15-76.2**	**2.23**	**9.4**	**1.31-66.9**
						
# animals (baseline: < 300)						
300-1 000	**2.37**	**10.7**	**1.34-86.2**	**2.34**	**10.4**	**1.52-71**
> 1 000	**2.95**	**19.1**	**2.53-145**	**3**	**20.2**	**3.13-130.4**
						
Scotland × 300-1 000	**-3.6**	**0.02**	**0-0.78**	**-3.6**	**0.02**	**0-0.57**
Scotland × > 1 000	**-4.3**	**0.01**	**0-0.2**	**-4.38**	**0.01**	**0-0.17**

Parameters in bold are significant at 0.05 level

## Discussion

This study attempted to assess the impact of the genotype distribution of scrapie-affected flocks on the prevalence of scrapie from a sub-population of the CSFS holdings. The selection of the holdings was based on three conditions: confirmation of classical scrapie by surveillance, its membership of the CSFS and the cull option: genotype & cull. Only sheep of types III and V were part of the cull population from which a sample was selected for testing, increasing the probability of detecting scrapie given the higher susceptibility to scrapie of animals with genotypes of these types.

The use of numeric covariates that represent proportions imposes certain restrictions to the statistical analyses that can be applied. The centred-log-ratio transformation allows the creation of the same number of variables in contrast to other methods like the additive-log-ratio [18] or the isometric-log ratio [22] transformations that produce one less than the original number of variables, making difficult the interpretation of the results. Moreover the new variables in the centred-log-ratio transformation are correlated with the original proportions so that increasing values of the *clr*-transformed variables represent increasing proportions of the corresponding compositional variables. Thus the overall results of the analysis concluded that an increase in the proportion of the ARR/AHQ, AHQ/VRQ, ARH/VRQ, ARQ/VRQ genotypes, in the proportion of NSP type V, or in the proportion of the ARH, ARQ, AHQ and VRQ alleles increased significantly the expected number of scrapie cases in affected holdings. On the contrary an increase in the proportion of the ARR/ARR genotype decreased significantly the expected number of scrapie cases.

The collation of genotype data using the date of death as the cut-off for inclusion may have introduced some errors. Some genotyped animals sent for slaughter before the initial cull were left in the final dataset, increasing the size of the study holdings. Although these animals may not have been in the holding at the time of the cull, they contributed to the genotype profile of the holding hence to the overall risk of the population to scrapie and to the spread of the disease within the flock. Had the farmers used this information to reduce the number of animals with susceptible genotypes in the flock, this inclusion would had introduced certain bias towards resistant genotypes, although only during a short period of time prior to the cull. Another reason for the large holding sizes is the aggregation of the count of sheep from different flocks within the same holding. The pooling of flocks within the same holding for culling and testing purposes prevents this study from considering the flock as the study unit.

Although the individual risk of scrapie is well determined by its genotype, the simple extrapolation to the population level would assume that the more susceptible animals a scrapie-affected flock has, the higher the prevalence of classical scrapie. However two major factors can render this assumption untenable: management and breeding practices and the overall genetic resistance of the flock. Modelling exercises have suggested that the genotype profile of a flock determines the overall size of an outbreak whereas the flock management practices determine the outbreak type [23]. If the management practices contributed to a heavily infected environment and to the horizontal transmission of the infection, only by measuring the biosecurity and management practices of each flock could this have been ascertained and tested. These potential epidemiological differences between holdings expressed by flock-level risk factors were not available and could not be included in the analysis.

This study attempted to address the genotype susceptibility/resistance since the dynamics of scrapie within flocks is better understood within the context of the genotype profile of the flock [9] rather than addressing the risk at the individual level. Although the genotype profile of a flock is dynamic depending on the breeding and replacement strategy [12], we attempted to capture the distribution of the genotypes in scrapie-affected flocks prior to the cull and testing following statutory measures. The results of the analysis using flock-level susceptibility risk indicators also showed the strong effect of the genotype profile of scrapie-affected holdings on the incidence of scrapie infection. Although measuring clinical disease, a previous study of 30 scrapie-affected flocks also showed that flocks of greater flock susceptibility tended to have larger outbreaks [8].

The negative association between ARR/ARR and the count of scrapie as in model 1 reinforces the efficacy at flock level of the breeding for resistance programmes aimed at increasing the frequency of the ARR allele in the general population. The results of this study are consistent with the findings of previous studies where the presence of the ARR allele reduces the risk of scrapie at the population level [5,24]. The abundance of animals in a flock with VRQ allele causes the opposite effect, followed by the decreasing effect of the ARQ, AHQ and ARH alleles in the overall incidence of the disease. The genotype frequencies of all cases confirmed by the different surveillance sources in GB between 2002 and 2008 also reflect the decreasing impact of the different alleles on the susceptibility to scrapie. Three genotypes, namely, ARQ/VRQ, ARQ/ARQ and VRQ/VRQ, account for 75% of all cases of classical scrapie confirmed during that period: all are included in the NSP types III and V [25]. According to the results of this study, genotypes of type V increase the risk of scrapie 3.5 times compared to NSP type I.

The positive effect of the ARR/AHQ allele is unexpected given the absence of classical scrapie in this genotype and the low susceptibility conferred by both alleles. This result does not have an apparent causal relationship and could have occurred by an undetected correlation with other variables, which has not been accounted for. Although speculative, the high prevalence of this genotype in Welsh affected holdings could be a confounding factor for high-risk farming practices leading to higher prevalence of infection.

The proportions of animals with susceptible genotypes in the sheep population of GB are very low as shown by the figures of the genotypes of the ram lamb population [26] and the genotype distribution of healthy populations [12], and so are in scrapie-affected flocks. The genotype with the lowest mean proportion in the study holdings is VRQ/VRQ (0.3%). This low frequency may be behind the fact that it did not appear in the final model 1. However when increasing its contribution in the form of NSP type V and the VRQ allele frequency as in models 2 and 3, these variables appeared significantly associated. The scarcity of this genotype could be due to the depletion due to clinical disease over the span of the epidemic in the flock. Epidemics of scrapie have been reported previously in flocks with hardly any VRQ/VRQ animals [9]. On the other hand it has been suggested that the frequency of the ARR allele in scrapie-affected flocks is lower that in the general population [11]. However the mean proportion of ARR/ARR animals in the study holdings (31.2%) and ARR allele frequency (54.1%) are higher than those reported by these authors in 15 scrapie-affected flocks with 17.4% and 41.3%, respectively, and those reported recently in a scrapie-affected flock in Italy with a frequency of the ARR allele of 23.5% [27]. With respect to the VRQ-associated genotypes, the frequency in the study population (4.7%) is much lower than the 15% of sheep in the flock described by Baylis et al. [9] but very similar to the 4.3% frequency of the VRQ-allele in the Italian flock.

The results of this study considering a large population of scrapie-affected holdings in Great Britain using the results of the statutory testing programme of the genotype and cull, confirmed that regardless the management practices under which the animals are kept, the increased susceptibility that the non-ARR alleles confer on an individual could be extrapolated at the population level. Increasing prevalence of ARR allele reduces the overall risk of scrapie at population level. The VRQ/VRQ genotype, present a very low frequency in the study population, seems to play a residual effect in the overall risk of scrapie in a flock.

## Competing interests

The authors declare that they have no competing interests.

## Authors' contributions

AO-P conceived the study, conducted the analysis and drafted the manuscript. JB extracted, cleaned and manipulated the genotype data for analysis. All authors read and approved the final manuscript.
